# Migrant blackbirds, *Turdus merula*, have higher plasma levels of polyunsaturated fatty acids compared to residents, but not enhanced fatty acid unsaturation index

**DOI:** 10.1002/ece3.6681

**Published:** 2020-08-17

**Authors:** Johan Kjellberg Jensen, Caroline Isaksson, Cas Eikenaar, Martin N. Andersson

**Affiliations:** ^1^ Department of Biology Lund University Lund Sweden; ^2^ Centre for Environmental and Climate Research (CEC) Lund University Lund Sweden; ^3^ Institute of Avian Research Wilhelmshaven Germany

**Keywords:** diet, fatty acids, migration, nutritional physiology, polyunsaturated fatty acids, *Turdus merula*

## Abstract

Birds have been observed to have dietary preferences for unsaturated fatty acids during migration. Polyunsaturated fatty acids (PUFAs) may increase the exercise performance of migrant birds; however, PUFAs are also peroxidation prone and might therefore incur increased costs in terms of enhanced oxidative damage in migratory individuals. To shed light on this potential constraint, we analyzed plasma fatty acid (FA) composition and estimated the unsaturation index as a proxy for susceptibility to lipid peroxidation of migrants and residents of the partially migratory common blackbird (*Turdus merula*) at a stopover site during autumn migration. As predicted, migrant birds had higher relative and absolute levels of PUFAs compared to resident birds. This included the strictly dietary ω‐3 PUFA α‐linolenic acid, suggesting a dietary and/or storage preference for these FAs in migrants. Interestingly, the FA unsaturation index did not differ between migrants and residents. These findings suggest a mechanism where birds alter their levels of metabolic substrate without simultaneously increasing the susceptibility of the substrate to lipid peroxidation. In summary, our results are in line with the hypothesis that increased exercise performance during migration might be constrained by oxidative stress, which is manifested in changes in the composition of key FAs to retain the unsaturation index constant despite the increased levels of peroxidizable PUFAs.

## Graphical abstract

Migrating blackbirds, *Turdus merula*, have higher plasma levels of exercise‐enhancing polyunsaturated fatty acids compared to resident conspecifics at the same stopover site. High levels of such unsaturated fatty acids may incur costs in terms of increased lipid peroxidation, and hence oxidative stress. Yet, the fatty acid unsaturation index did not differ between migrants and residents, suggesting physiological mechanisms in migrants to mitigate this potential cost.

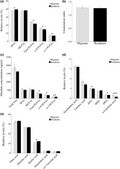

## 1 INTRODUCTION

The costs associated with migratory flight cause birds to optimize their food intake and utilize specific nutritional compounds (Lindström, 1991; Pierce & McWilliams, 2014). Fat is an important source of fuel for migrating birds, as it represents the most efficient way to store biochemical energy (McWilliams, Guglielmo, Pierce, & Klaassen, 2004). Of the different types of fat, fatty acids (FAs) with a higher level of unsaturation have higher mobilization rates than other FAs and might therefore be preferentially oxidized by birds during migration (McWilliams et al., 2004; Price, Krokfors, & Guglielmo, 2008). Indeed, birds generally seem to store more unsaturated FAs in their adipose tissue while migrating (Blem, 1976; Johnston, 1973)**;** however, the physiological function and composition of specific FAs during migration is still unclear and may be species‐dependent or differ between long‐ and short‐distance migrants.

Although saturated FAs (SFAs) and monounsaturated FAs (MUFAs) can be biosynthesized *de novo* by vertebrates, the production of long‐chained polyunsaturated FAs (PUFAs) requires dietary intake of the essential ω‐6 PUFA linoleic acid and the ω‐3 PUFA α‐linolenic acid. However, long‐chain PUFA biosynthesis can be inefficient (Klasing, 1998; Sanders, 1988), and several studies of birds have shown that the diet influences the PUFA composition of their tissues, including fat stores, muscles, liver, and plasma (Andersson, Nilsson, Nilsson, & Isaksson, 2018; Andersson, Wang, Nord, Salmón, & Isaksson, 2015; Ben‐Hamo, Mccue, Mcwilliams, & Pinshow, 2011; Maillet & Weber, 2006; Pierce & McWilliams, 2005; Pierce, McWllliams, Place, & Huguenin, 2004; Price & Guglielmo, 2009). Therefore, a dietary preference for unsaturated FAs observed in migratory bird species would not be surprising. Indeed, both yellow‐rumped warblers (*Setophaga coronata*) and red‐eyed vireos (*Vireo olivaceus*) caught during migration preferred diets enriched with either PUFAs or MUFAs over SFAs, when investigating FAs with up to 18‐carbon chain lengths (McWilliams, Kearney, & Karasov, 2002; Pierce et al., 2004). Although these studies lacked a nonmigratory control group of the same species, and thus offer no clear link to the migratory behavior in itself, they demonstrate a dietary preference for these unsaturated FAs and suggest that migrating bird species are able to distinguish between food items based on FA content.

Enhancement of exercise performance has been proposed to be associated with a PUFA‐rich diet, which may be particularly relevant for migratory birds and could explain the potential dietary preference (Pierce & McWilliams, 2014; Pierce, McWilliams, O’Connor, Place, & Guglielmo, 2005; Price & Guglielmo, 2009). However, studies to date report different results across species, performance measurements, and the age of the birds (e.g., Dick & Guglielmo, 2019a; McCue, Amitai, Khozin‐Goldberg, McWilliams, & Pinshow, 2009; McWilliams & Pierce, 2006; Pierce et al., 2005; Price et al., 2018). For example, red‐eyed vireos (*V. olivaceus*) and white‐throated sparrows (*Zonotrichia albicollis*) that were fed ω‐6 PUFAs had higher peak metabolic rates compared to birds fed MUFAs or ω‐3 PUFAs, respectively (Pierce et al., 2005; Price & Guglielmo, 2009). However in starlings (*Sturnus vulgaris*), birds provided a diet rich in ω‐6 PUFA (linoleic acid) decreased energy expenditure, fat metabolism, as well as basal and peak metabolism compared to birds that were fed a low ω‐6 PUFA diet (Carter, DeMoranville, Pierce, & McWilliams, 2020). It was suggested that the signaling property of linoleic acid is the mechanism behind this metabolic change (Carter et al., 2020). Similarly, in the study of Klaiman, Price, and Guglielmo (2009) linoleic acid was pointed out to play a key role in seasonal variation of FAs in white‐throated sparrows. Other studies have focused on the intake of long‐chained ω‐3 PUFAs and a hypothesized “doping effect” of these PUFAs during migration (e.g., Maillet & Weber, 2006, 2007; Nagahuedi, Popesku, Trudeau, & Weber, 2009). In line with this hypothesis, semipalmated sandpipers (*Calidris pusilla*) prepare for migration by feeding on marine invertebrates containing high amounts of the long‐chained ω‐3 PUFAs eicosapentaenoic acid (EPA) and docosahexaenoic acid (DHA) (Maillet & Weber, 2006). These ω‐3 PUFAs are incorporated into the flight muscle of the sandpiper and affect the activity of oxidative enzymes in the muscle (Maillet & Weber, 2007), which in turn was demonstrated to affect aerobic metabolism in bobwhite quails (*Colinus virginianus*) (Nagahuedi et al., 2009). However, in the yellow‐rumped warbler (*S. coronata*), the relative abundance of both ω‐3 PUFAs and ω‐6 PUFAs did not affect flight performance (Dick & Guglielmo, 2019a). Thus, the effects of FAs on exercise performance in birds are still not fully understood, and further studies are needed to better understand the role of PUFAs in bird migration.

Increased PUFA levels comes with a potential cost, since migration exposes birds to high levels of reactive oxygen species (ROS) as a consequence of the long‐distance flight (Eikenaar, Hegemann, Packmor, Kleudgen, & Isaksson, 2020; Jenni‐Eiermann, Jenni, Smith, & Costantini, 2014). Paradoxically, the molecular structure linked to exercise enhancement, PUFAs’ multiple double bonds, also makes them prone to peroxidation by the ROS, increasing the risk of oxidative stress (Hulbert, Pamplona, Buffenstein, & Buttemer, 2007). Migratory white‐throated sparrows (*Z. albicollis*) fed with PUFAs (both α‐linolenic and linoleic acid) showed an increase in oxidative damage (Alan & McWilliams, 2013) and similar results have been found also in other species (Labbe, Trick, & Bearerogers, 1991; Sies, Stahl, & Sevanian, 2005, but see also; Andersson et al., 2018; Dick & Guglielmo, 2019b). Comparable to the dietary preference for unsaturated FAs, birds may also consume more fruits with high antioxidant levels at stopover sites during migration (Bolser et al., 2013). Indeed, as the main energy source for migratory flight is fat, with a likely preference for unsaturated FAs (McWilliams et al., 2002; Pierce et al., 2004), an increased antioxidant defense might be especially important to shield against peroxidation of migrants’ cell membranes or fuel deposits.

The aim of this study was to investigate FA variation in relation to migration in a partially migrating species, the common blackbird *Turdus merula* (blackbird hereafter), and analyze whether a potential difference in PUFA content may affect the overall unsaturation level of circulating lipids. Our natural study system allowed us to investigate, for the first time, FA composition in wild birds with either a resident or migratory status, at the same location and time. Specifically, we predicted that migrant birds should have higher relative and absolute levels of ω‐6 PUFAs, ω‐3 PUFAs, and hence total PUFAs, compared to resident birds. Since previous studies on migratory bird species have reported dietary preferences based on FA content, we also predicted that migrant blackbirds would differ from residents in their levels of essential FAs (linoleic‐ and α‐linolenic acid). In addition, by estimating a FA unsaturation index, we tested whether the predicted increase in PUFAs would increase the susceptibility of the circulating FA substrate to lipid peroxidation.

## 2 MATERIALS AND METHODS

### 2.1 Field methods and sampling

Blackbirds were trapped and sampled on the small (<1 km^2^) and isolated island of Helgoland (54°11′N, 07°55′E) in the North Sea, off the coast of Germany. The sampling took place during autumn migration in October 2014, when hundreds of birds use the island as a stopover site, mixing with the local resident blackbird population (Dierschke, Dierschke, Hüppop, Hüppop & Jachmann, 2011). Most of the migrant blackbirds stopping over on the island winter in the UK and breed in Scandinavia (Dierschke et al., 2011).

Blood samples were taken from the birds within 10 min of capture, by puncturing a brachial vein. The samples were kept on ice until plasma was separated by centrifuging the samples. This was done as soon as possible and no later than 4 hr after capture. The plasma samples were stored at −20°C, for a maximum of a month, until further analysis. All captured individuals were aged (1st year or adult) and sexed based on plumage (Svensson, 1992), and visually scored for body fat on Kaiser's (1993) scale from 0 (no fat) to 8 (furcular and abdomen bulging, and breast covered with fat). All trapping took place between 7 a.m. and 6 p.m. and was approved by the Ministry for Agriculture, the Environment, and Rural Areas, Schleswig‐Holstein, Germany.

Assignment of the birds’ status (migrant or resident) was done following Eikenaar, Isaksson, and Hegemann (2018). All birds were color‐ringed in addition to receiving a metal ring. Searches for color‐ringed birds were carried out throughout October and November. Due to the very small size of the island (<1 km^2^), the birds were easily sighted if they remained in the area. Recent data show that the longest migratory stopover observed in blackbirds on Helgoland was 20 days (5 days being the median duration), and thus, 20 days was used as a cutoff for determining the migratory status of the birds (Packmor, Klinner, Woodworth, Eikenaar, & Schmaljohann, 2020).

Birds observed again 20 days or more after color ringing were assigned the resident status. Resident birds were frequently resighted or retrapped, with eight observations being the median of all assigned resident birds. In addition, blackbirds ringed (with a metal ring only) on Helgoland in previous breeding seasons and retrapped by us in autumn were assumed to be Helgoland residents. This assumption is based on a radio‐telemetry study showing that 91% of the Helgoland blackbirds are sedentary (Sacher, 2009). The assumption seems valid since nine of the ten birds from this category were resighted on Helgoland several weeks after we color‐ringed them (the one exception was resighted only once, after eight days). In total, 26 birds were assigned the resident status. Note that each individual was only sampled for blood at first trapping in autumn, even if some birds were retrapped several times. Also, the number of days spent by migrating birds on the island prior to sampling was unknown. A total of 42 individuals caught and color‐ringed, but never resighted were assigned the migratory status. In a number of cases, birds were resighted within the 20‐day window, but not after, and were thus likely migrants. However, keeping a conservative classification, these individuals were excluded from the study to minimize the risk of miss‐classifying the migratory status.

### 2.2 Fatty acid extraction and analysis

The extraction and analysis of FAs followed Andersson et al. (2018), using 5 μl of plasma. FAs were extracted using 50 µl chloroform:methanol (2:1 v/v) containing 1.67 μg/μl methyl *cis*‐10‐heptadecenoate (>99% pure, Aldrich) as an internal standard. The FAs were then transformed into FAMEs (fatty acid methyl esters) through base methanolysis using 100 µl of 0.5 M KOH/Me (1 hr at 40°C). The reaction was terminated using 100 µl of 0.5 M HCl/Me, and FAMEs were extracted in 300 µl *n‐*heptane (>99% pure, VWR Prolabo). Samples were analyzed using an Agilent 5975 mass spectrometer (MS) coupled to an Agilent 6890 gas chromatograph (GC), which was equipped with an HP‐INNOWax PEG column (30 m, 0.25 mm i.d., 0.25 mm film thickness; Agilent). Helium was used as carrier gas at a constant flow of 1 ml/min. The GC oven temperature was programmed to 80°C for 1 min and then increased by 10°C/min to 230°C and held for 20 min. The chromatograms were analyzed using Agilent ChemStation software. The FAMEs were identified by comparison of retention times and mass spectra of known synthetic standards (Supelco 37 Component FAME mix, Sigma‐Aldrich).

In total, 20 FAs were quantified through their methyl ester derivatives. The proportion of each FA per individual was calculated by dividing the peak area for individual FAs with the sum of all FA peak areas. These relative levels were then logit transformed $\left(\log\;\left(\frac{y}{1‐y}\right)\right)$ before statistical analysis (Warton & Hui, 2011).

### 2.3 Statistical analyses

Before statistical analysis, the individual PUFAs, MUFAs, and SFAs were pooled into total PUFAs, total ω‐3 PUFAs, total ω‐6 PUFAs, total MUFAs, and total SFAs, respectively. In addition, to better understand the details of the physiological differences, each FA present in the plasma at an average relative level > 1% in either migrants or residents (or both groups) was also analyzed separately (Hulbert & Abbott, 2011; Klasing, 1998; Pierce & McWilliams, 2014), including palmitic acid [16:0], stearic acid [18:0], palmitoleic acid [16:1n‐7], *cis*‐vaccenic acid [18:1n‐7], oleic acid [18:1n‐9], α‐linolenic acid [18:3n‐3], EPA [20:5n‐3], docosapentaenoic acid [DPA; 22:5n‐3], DHA [22:6n‐3], linoleic acid [18:2n‐6], and arachidonic acid [20:4n‐6].

Statistical analysis was conducted using R version 3.5.1 (R Core Team, 2018). Generalized linear models (GLMs) were used to investigate the difference in FA composition between the migratory and resident birds. We used a hypothesis driven approach, with the statistical models kept the same for all FAs and FA groups, facilitating comparisons of the effects across the different FAs. The models included migratory status as a fixed factor and time of sampling and body fat score as covariates. Time of sampling was included since it is established that daily feeding regimes and metabolism influence circulating FA levels and proportions (Isaksson, Hanson, & Burdge, 2015). To further explore if any time of sampling effect differed between migrants and residents, we also tested the interaction between time of sampling and migration status. Time of sampling was mean centered to facilitate interpretation of regression coefficients. Age and sex were not included in the final models, as no general effect of these factors was found, and also to simplify the models (the results from the models with sex and age included are reported in Appendix S2a–c). In total, 17 GLMs were performed with the relative levels of pooled and single FAs as response variables. Six additional GLMs were carried out with the absolute concentrations (ng FA/μl plasma) of the pooled FA groups, including total FA concentration as the response variable. As a final response variable, the unsaturation index was estimated. The degree of unsaturation was calculated as an index of the sum of each FA’s relative plasma level, multiplied with their respective number of double bonds (Jezierska, Hazel, & Gerking, 1982; McCue, 2008).

To complement the hypothesis testing based on the GLMs, the Hedges’ *g* standardized unbiased effect size (Hedges & Olkin, 1985) was calculated for each FA proportion by dividing the difference of the group means by the pooled standard deviation of the entire population. The effect size is thus a measurement of the magnitude of the quantitative difference (i.e., biological effect) between the groups and is used to standardize differences between means (Kelley & Preacher, 2012; Nakagawa & Cuthill, 2007). An effect size value of 0.5 is generally considered to indicate a medium effect and a value of 0.8 or above a large effect (Cohen, 1992).

## 3 RESULTS

**FIGURE 1:**
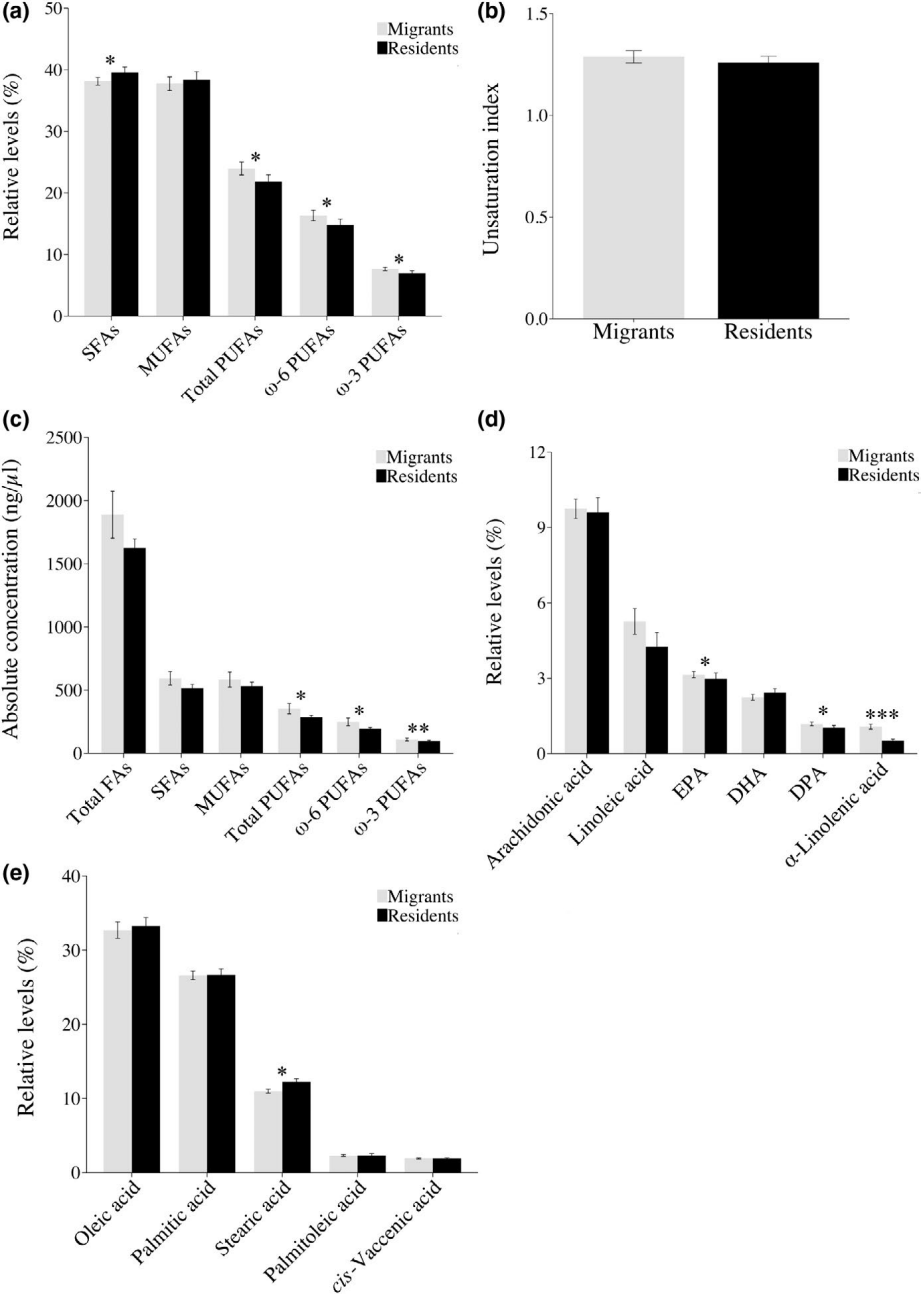
Fatty acid (FA) profiles in blood plasma of migrant and resident blackbirds. Relative levels (percent of total FA content) of (a) pooled FA groups, (b) unsaturation index (see Methods for details), (c) absolute concentrations of circulating FAs (ng/μl), (d) Relative levels (percent of total FA content) of individual PUFAs, and (e) individual SFAs and MUFAs in migrant (gray) and resident (black) blackbirds. All panels show mean ± standard error (SE). Significance levels are indicated by asterisks (*p = .05–0.01, **p = .01–0.001, ***p < .001). DHA, docosahexaenoic acid; DPA, docosapentaenoic acid; EPA, eicosapentaenoic acid; MUFAs, monounsaturated fatty acids; PUFAs,  polyunsaturated fatty acids; SFAs, saturated fatty acids

In total, 20 FAs were identified in the blackbird plasma samples (Table 1), including ten PUFAs, seven MUFAs, and three SFAs. The relative level of total PUFAs was higher in migrant compared to resident birds (*F*_1, 63_ = 5.441, *p* = .023; Figure 1a; see Appendix S1a for additional statistical details). A similar difference was found for total ω‐3 PUFAs (*F*_1, 63_ = 7.391, *p* = .008) and for total ω‐6 PUFAs (*F*_1, 63_ = 4.056, *p* = .048). Of the individual ω‐3 PUFAs analyzed, α‐linolenic acid differed depending on migration status, with higher relative levels in migrant birds (*F*_1, 63_ = 21.138, *p* < .0001; Figure 1d; Appendix S1b). Also, DPA (*F*_1, 63_ = 5.297, *p* = .025) and EPA (*F*_1, 63_ = 6.821, *p* = .011) were higher in the migrants than in the residents, but DHA showed no difference (*F*_1, 63_ = 0.133, *p* = .716). The two individually analyzed ω‐6 PUFAs did not differ between migrants and residents, although arachidonic acid showed a trend (arachidonic acid: *F*_1, 63_ = 3.928, *p* = .052; linoleic acid: *F*_1, 63_ = 1.370, *p* = .246).

**TABLE 1:** Relative levels (percent of total fatty acid content) of all identified circulating fatty acids in resident and migratory blackbirds

Fatty acid	Migrants			Residents		
	*N*	Mean (%)	*SE*	*N*	Mean (%)	*SE*
Myristic acid	42	0.55	0.084	26	0.63	0.123
14:0 (SFA)						
Myristoleic acid	42	0.05	0.007	26	0.07	0.014
14:1n‐5 (MUFA)						
Palmitic acid	42	26.60	4.105	26	26.65	5.226
16:0 (SFA)						
Palmitoleic acid	42	2.30	0.355	26	2.31	0.453
16:1n‐7 (MUFA)						
16:1n‐9 (MUFA)	42	0.32	0.049	26	0.39	0.077
Stearic acid	42	10.98	1.695	26	12.27	2.407
18:0 (SFA)						
*cis*‐Vaccenic acid	42	1.93	0.298	26	1.91	0.375
18:1n‐7 (MUFA)						
Oleic acid	42	32.71	5.047	26	33.26	6.522
18:1n‐9 (MUFA)						
Linoleic acid	42	5.26	0.812	26	4.25	0.833
18:2n‐6 (ω‐6 PUFA)						
α‐Linolenic acid	42	1.07	0.097	26	0.52	0.067
18:3n‐3 (ω‐3 PUFA)						
Paullinic acid	42	0.18	0.028	26	0.23	0.045
20:1n‐7 (MUFA)						
Gondoic acid	42	0.26	0.040	26	0.21	0.041
20:1n‐9 (MUFA)						
Eicosadienoic acid	42	0.59	0.053	26	0.35	0.024
20:2n‐6 (ω‐6 PUFA)						
dihomo‐γ‐Linolenic acid	42	0.43	0.023	26	0.37	0.030
20:3n‐6 (ω‐6 PUFA)						
Arachidonic acid	42	9.74	0.383	26	9.61	0.572
20:4n‐6 (ω‐6 PUFA)						
Eicosapentaenoic acid (EPA)	42	3.14	0.121	26	2.98	0.229
20:5n‐3 (ω‐3 PUFA)						
Osbond acid	42	0.31	0.047	26	0.26	0.050
22:5n‐6 (ω‐6 PUFA)						
Docosapentaenoic acid (DPA)	42	1.18	0.070	26	1.04	0.088
22:5n‐3 (ω‐3 PUFA)						
Docosahexaenoic acid (DHA)	42	2.24	0.346	26	2.42	0.475
22:6n‐3 (ω‐3 PUFA)						

The relative level of total MUFAs did not differ between migrant and resident birds (*F*_1, 63_ = 0.549, *p* = .461; Figure 1a; Appendix S1a) and neither did oleic acid (*F*_1, 63_ = 0.454, *p* = .503; Figure 1e; Appendix S1b), palmitoleic acid (*F*_1, 63_ = 0.012, *p* = .913) nor *cis*‐vaccenic acid (*F*_1, 63_ = 0.181, *p* = .672). The relative level of total SFAs was significantly lower in migrant compared to resident birds (*F*_1, 63_ = 4.049, *p* = .048; Figure 1a). The same pattern was shown for stearic acid (*F*_1, 63_ = 5.364, *p* = .024; Figure 1e), but not for palmitic acid (*F*_1, 63_ = 0.957, *p* = .332).

In addition to relative levels, the absolute FA concentrations (ng/μl plasma) of all pooled FA groups were analyzed (Figure 1c; Appendix S1c). The total concentration of circulating FAs did not differ between the groups, although a trend for higher levels in migrants was evident (*F*_1, 63_ = 3.484, *p* = .067). The concentration of total PUFAs differed between migrant and resident birds, with migrants having higher concentrations (*F*_1, 63_ = 5.892, *p* = .018). Concentrations of both PUFA sub‐groups showed similar differences (ω*‐*3 PUFAs: *F*_1, 63_ = 8.175, *p* = .006; ω*‐*6 PUFAs: *F*_1, 63_ = 5.610, *p* = .021). The concentrations of total MUFAs and total SFAs did not differ between migrant and resident birds (MUFAs: *F*_1, 63_ = 2.615, *p* = .111; SFAs: *F*_1, 63_ = 1.950, *p* = .168; Figure 1c). Interestingly, the unsaturation index did not differ between migrants and residents (*F*_1, 63_ = 0.468, *p* = .497; Figure 1b).

**FIGURE 2:**
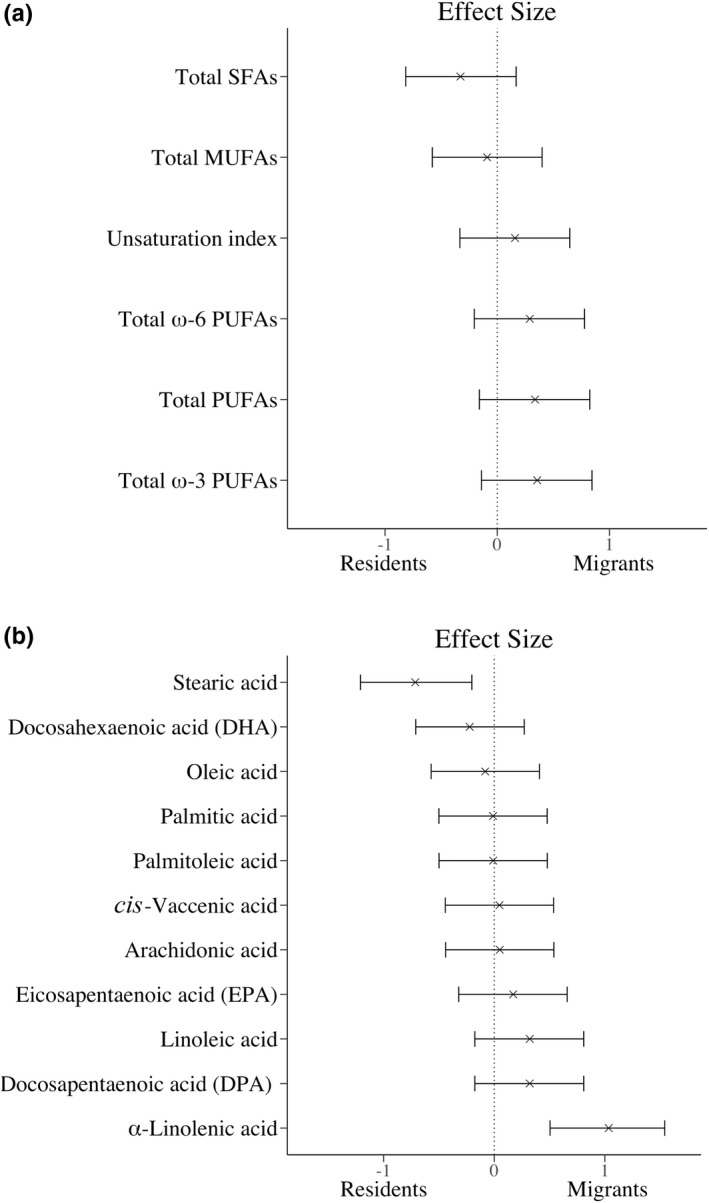
Effect sizes for fatty acids (FAs) in migrant and resident blackbirds. Hedges g standardized unbiased effect size of (a) pooled fatty acid (FA) groups and (b) individual FAs for migrant and resident blackbirds. The effects sizes are ordered from lowest to highest g‐value. Positive values indicate higher proportions in migrants, while negative values indicate lower proportions in migrants. Only FAs with relative levels > 1% of total FA content are shown. Bars display the 95% confidence intervals. MUFAs, monounsaturated fatty acids; PUFAs, polyunsaturated fatty acids; SFAs, saturated fatty acids

The effect sizes for relative levels of all pooled and individual FAs are shown in Figure 2a,b, respectively. None of the pooled FAs groups had a medium effect or above (effect size greater than g = 0.5), with total ω‐3 PUFAs being the highest at g = 0.36 (Figure 2a). Of the single FAs, the ω‐3 PUFA α‐linolenic acid was associated with the largest effect (g = 1.03; Figure 2b).

The relative levels the PUFAs DHA (*F*_1, 63_ = 7.986, *p* = .006) and arachidonic acid (*F*_1, 63_ = 5.656, *p* = .020; Appendix S1b) showed a negative correlation with time of sampling. The relative level of total SFAs increased over the day (*F*_1, 63_ = 4.235, *p* = .044) and so did palmitic acid (*F*_1, 63_ = 8.419, *p* = .005). Absolute concentrations of total PUFAs (*F*_1, 63_ = 4.539, *p* = .037), ω‐3 PUFAs (*F*_1, 63_ = 4.485, *p* = .038), and ω‐6 PUFAs (*F*_1, 63_ = 4.077, *p* = .048) all decreased over the day (Appendix S1c). In all other cases, time of sampling did not affect the FA levels.

**FIGURE 3:**
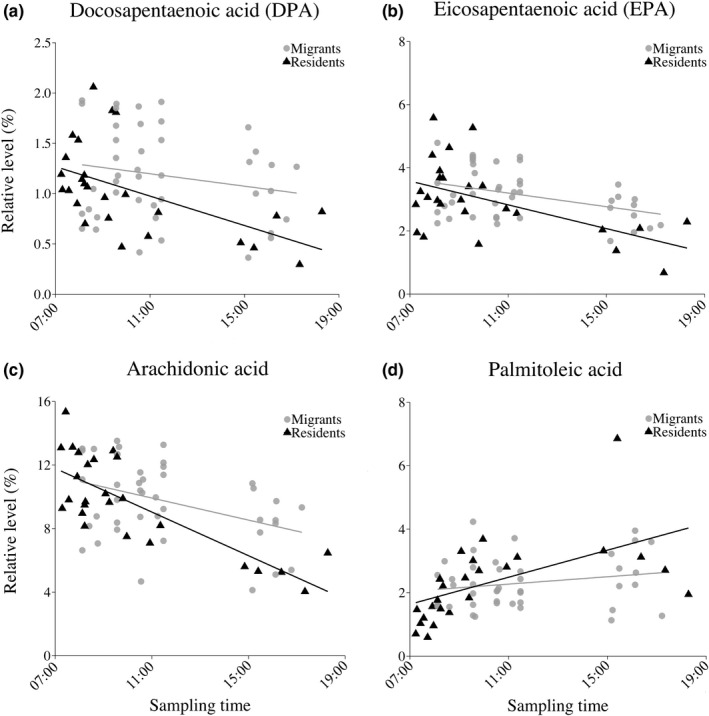
Relative levels of the four fatty acids (FAs) showing a significant interaction between migration status and time of sampling. (a) Docosapentaenoic acid (DPA), (b) eicosapentaenoic acid (EPA), (c) arachidonic acid, and (d) palmitoleic acid, where gray circles indicate migrant individuals and black triangles resident individuals

The relative levels of the long‐chained PUFAs arachidonic acid, EPA, and DPA showed a significant interaction between migration status and time of sampling (arachidonic acid: *F*_1, 63_ = 8.354, *p* = .005; EPA: *F*_1, 63_ = 4.950, *p* = .030; DPA: *F*_1, 63_ = 5.139, *p* = .027; Figure 3a–c; Appendix S1b). Such an interaction was also present for the relative levels of the MUFA palmitoleic acid (*F*_1, 63_ = 6.502, *p* = .013, Figure 3d), but not for other FAs. The slopes for DPA, EPA, and arachidonic acid showed a steeper decline with time of sampling for resident compared to migrant birds. In contrast, residents had a steeper increase in palmitic acid with time of sampling compared to migrants.

Body fat score did not differ between the migrant and resident birds (t_66_ = 0.171, *p* = .865). Relative levels of PUFAs declined with increasing body fat score (total PUFAs: *F*_1, 63_ = 10.216, *p* = .002, total ω‐3 PUFAs: *F*_1, 63_ = 12.841, *p* < .001, and total ω‐6 PUFAs: *F*_1, 63_ = 8.413, *p* = .005; Appendix S1a). The relative levels of individual PUFAs revealed similar patterns as the pooled relative PUFA groups (α‐linolenic acid: *F*_1, 63_ = 12.085, *p* < .001; DPA: *F*_1, 63_ = 7.074, *p* = .010; arachidonic acid: *F*_1, 63_ = 9.496, *p* = .003; and linoleic acid: *F*_1, 63_ = 4.377, *p* = .040; Appendix S1b). In addition, relative levels and absolute concentrations of MUFAs increased with fat score (relative levels: *F*_1, 63_ = 11.665, *p* = .001; absolute concentration: *F*_1, 63_ = 6.228, *p* = .015). This was also the case for the relative level of oleic acid (*F*_1, 63_ = 12.331, *p* < .001). For all other FAs, body fat score was not significant.

## 4 DISCUSSION

### 4.1 Fatty acid composition and unsaturation indices in relation to migratory status

The preferential ingestion, storage, and mobilization of certain fatty acids during energy demanding periods such as migration or development have the potential to enhance performance, ultimately affecting fitness (e.g., Maillet & Weber, 2006; McWilliams et al., 2004; Pierce & McWilliams, 2014; Pierce et al., 2005; Twining et al., 2016). Yet, the extent to which this occurs in the wild, and whether a potential risk of enhanced oxidative cost is linked to the predicted increase in FA unsaturation via intake of PUFAs, is still not fully understood, with variable results reported from different experiments (Alan & McWilliams, 2013; Dick & Guglielmo, 2019b). Here, we addressed this question in a partial migrant, the common blackbird.

In accordance with our prediction, migrating blackbirds had higher relative and absolute levels of PUFAs compared to resident conspecifics. This could be due to physiological and behavioral differences related to their migratory status and/or to dietary differences independent of migratory status, such as feeding ecologies at the breeding grounds. The migratory individuals, however, originate from several different locations (Dierschke et al., 2011), suggesting that diet availability at breeding grounds is unlikely to be the only explanation for the observed differences in PUFA levels between migrants and residents. Thus, it is likely that PUFA‐rich food items are preferentially eaten by migrants (after or prior to the arrival at the stopover site) or that they eat larger quantities of PUFA‐rich food, and/or oxidize the PUFAs differently, which is reflected in the circulation during migration. Previous studies have shown differences in adipose tissue composition depending on temporal migration status (Johnston, 1973; Maillet & Weber, 2006; Pierce & McWilliams, 2005), yet circulating FAs have received little attention, especially in a natural setting (but see Guglielmo, Williams, Zwingelstein, Brichon, & Weber, 2002).

Migrant blackbirds had twice as high relative levels of α‐linolenic acid compared to resident birds, with an especially large effect size, although this FA accounted for a low proportion of the total plasma FA content (1.07% and 0.52% in migrants and residents, respectively). This ω‐3 PUFA cannot be biosynthesized by vertebrates (Sanders, 1988), which suggests that dietary preferences (i.e., food types) of blackbirds relate to migration status. Seeds and terrestrial invertebrates, such as earthworms, contain α‐linolenic acid and could hence represent the dietary sources of this FA (Andersson et al., 2018; Beare‐Rogers, Dieffenbacher, & Holm, 2001; Hansen & Czochanska, 1975; Isaksson et al., 2015). The differences were smaller for the long‐chained ω‐3 PUFAs EPA and DPA, but their plasma proportions were still also significantly higher in migrants than residents, suggesting that migrants overall ingest or release more ω‐3 PUFAs from storages or that they biosynthesize these PUFAs from dietary α‐linolenic acid. Although there was no significant difference in the proportions of individual ω‐6 PUFAs between migrants and residents, the proportion of total ω‐6 PUFA was slightly higher in migrants, driven by the longer‐chained arachidonic acid, which showed a trend toward significance. ω‐3 PUFAs were previously suggested to be incorporated in the membrane of muscle cells in the migrating semipalmated sandpiper to boost their flight performance (Maillet & Weber, 2006). For other migratory species, however, ω‐6 PUFAs rather than ω‐3 PUFAs seem to enhance exercise performance such as for the white‐throated sparrow (Price & Guglielmo, 2009). Furthermore, recent studies on migratory yellow‐rumped warblers revealed larger diet‐induced differences between treatment groups for total ω‐3 PUFA content in muscle phospholipids as compared to the differences observed in the present study, but without any effect on flight performance (Dick & Guglielmo, 2019a). It remains unclear whether the comparatively small differences in ω‐3 and ω‐6 PUFAs result in increased flight performance of migratory blackbirds. However, given that blackbirds migrate rather short distances, a small increase in PUFA levels may already suffice to boost flight performance; alternatively, the small differences may indicate that increasing the PUFA content is of limited relevance for short‐distance migrants. Irrespectively, as long as the costs of optimized PUFA intake (e.g., food search costs) are lower than the benefits of increased migratory performance, there will be an evolutionary pressure to optimize PUFA intake. Regardless, our results show that migrant blackbirds circulate higher levels of PUFAs, especially ω‐3 PUFAs (except DHA), compared to resident birds sampled at the same location and time.

Migrant blackbirds had lower relative levels of SFAs, yet they showed a weak trend (*p* = .168) for higher absolute concentration of SFAs, compared to resident birds. Given that SFAs are highly abundant in the plasma, this could contribute to the near‐significant (*p* = .067) trend for overall higher concentrations of total circulating FAs in migrant birds. In turn, this could be the result of increased feeding activity but also increased *de novo* biosynthesis of SFAs. For example, migratory black‐tailed godwits (*Limosa limosa*) were shown to upregulate *de novo* lipogenesis when feeding from fat‐poor diets (rice seeds) (Araujo et al., 2019; Viegas et al., 2017). Indeed, since the migrating blackbirds were observed to incorporate fat‐poor berries in their diets (Dierschke et al., 2011), increased *de novo* lipogenesis may contribute to the observed trend for higher absolute SFA concentrations in the migrants.

Despite the difference in the levels of total PUFAs, no significant difference was found between migrants and residents in their unsaturation index. Similarly to the present study, Eikenaar, Källstig, Andersson, Herrera‐Duenas, and Isaksson (2017) did not find a difference in peroxidation index (i.e., a similar index as the unsaturation index, but in this case based on absolute FA concentrations in plasma) between migrant and resident blackbirds from the same study site, although a trend was evident. One potential cost of higher levels of PUFAs, and thus increased FA unsaturation, is an increased risk of lipid peroxidation, which migrating birds are particularly exposed to as endurance flights cause increased generation of ROS (Costantini, Cardinale, & Carere, 2007; Hulbert et al., 2007; Jenni‐Eiermann et al., 2014). Interestingly, and in line with the lack of difference in unsaturation index, the migrant and resident blackbirds in Eikenaar et al. (2017) did not differ in plasma lipid peroxidation (MDA); however, the migrants had higher nonenzymatic antioxidant levels. Further adding to the scenario is the observed lack of difference in the most unsaturated and peroxidation prone of all FAs, namely DHA, the only ω‐3 PUFA that was not higher in the migrants, but rather showed a trend for higher levels in residents. The similar unsaturation indices between migration strategies can in fact be largely explained by the lower, although nonsignificant, relative levels of DHA and the abundant MUFA oleic acid. This in itself is intriguing as diet choice experiments on other migratory bird species did not find a clear preference for either MUFAs or PUFAs, but rather for unsaturation at large (McWilliams et al., 2002; Pierce et al., 2004). This suggests that migrating birds, at least blackbirds, may retain higher plasma levels of PUFAs that might enhance exercise performance while mitigating the risk of increased peroxidation of circulating lipids by regulating their FA unsaturation index. In white‐throated sparrows, a PUFA‐rich diet increased oxidative damages to hydroperoxides (which can be derived from fatty acids, dROM) in the plasma, although lipid peroxidation index was not estimated (Alan & McWilliams, 2013). Possibly an experimental increase in PUFAs makes it difficult to retain the unsaturation level, hence the increase in damages. Another study reported no effects of ω‐3 or ω‐6 PUFA‐rich diets on oxidative damages to proteins in flight muscles at rest and after endurance flight in yellow‐rumped warblers (Dick & Guglielmo, 2019b). Although lipid peroxidation was not measured, the lack of increased protein damage suggests that PUFA diet did not increase oxidative stress overall.

Trade‐offs involving oxidative stress have been suggested for migratory birds before. For example, in a study using the same blackbird system, a negative correlation was found between nonenzymatic antioxidant capacity and microbial killing capacity in migrant blackbirds, but not in residents (Eikenaar et al., 2018). This could indicate that migratory individuals trade‐off immune function with antioxidant defenses during migration (Eikenaar et al., 2017, 2018). As the main energy source for migratory flight is fat, with a hypothesized preference for peroxidizable unsaturated FAs, especially PUFAs, in migratory species (McWilliams et al., 2002; Pierce et al., 2004), the increased antioxidant defense might be especially important to shield against peroxidation of migrants’ cell membranes or fuel deposits. Furthermore, migratory birds have been suggested to preferentially feed on antioxidant rich food during stopovers (Bolser et al., 2013), indicating that migrating birds optimize their nutritional physiology for the demanding journey (Pierce & McWilliams, 2014). Most likely, exercise performance gains and peroxidation risk would be key factors in determining a potential optimal FA composition for migration.

### 4.2 Daily variation in fatty acids in relation to migratory status

Daily changes in circulating FAs are expected due to circadian‐dependent feeding patterns along with changed metabolic processes and requirements over the course of the day. Some of these daily changes may depend on the migratory status because of differences in behaviors and physiological preparations for migration. Indeed, daily variation in long‐chained PUFAs was dependent on migration status, with a steeper decline over the day for arachidonic acid, EPA, and DPA in residents compared to migrant blackbirds. The MUFA palmitoleic acid showed the opposite trend, with a steeper increase for resident blackbirds. Among the three long‐chained PUFAs with a status‐dependent time of sampling effect, only arachidonic acid showed a decline over the day for both migrants and residents. A time‐dependent decline over the day was seen for the relative levels of the long‐chained DHA independent of migratory status. The decrease over the day in long‐chained PUFAs has also been reported in great tits, *Parus major* (Isaksson et al., 2015). The overall decline with time of sampling could indicate that the metabolic conversion of α‐linolenic acid and linoleic acid to longer‐chained PUFAs is, as previously suggested, not that rapid and efficient, and the relative concentrations of longer‐chained PUFAs only increase during the postabsorptive state (Klasing, 1998; Sanders, 1988). However, the difference in the slopes between the two status groups suggests that migrating birds retain relatively higher levels of three out of four long‐chained PUFAs throughout the day, possibly to have them readily available for a potential upcoming nocturnal flight. This is despite the fact that absolute levels of PUFAs decreased over the day. Furthermore, among the SFAs only the relative level of palmitic acid changed with time. With palmitic acid being the most abundant SFA, it was not surprising that total SFA also increased over the day. Since palmitic acid is a shorter‐chained SFA (16:0), lipogenesis could play a role in the increased relative levels over the day.

We observed no effect of time of sampling on absolute levels of total circulating FAs. This is somewhat surprising but indicates that a constant and stable circulation of FAs is maintained despite that food of varying FA content is taken in and absorbed throughout the day. Although there was considerable variation among the blackbirds in the absolute concentration of FAs (approx. 1,600–2,100 ng/μl), there is likely a threshold for a maximum level as a result of physiological limitations, for example, viscosity of the blood and uptake in the intestine. Thus, adjusting the relative levels of certain FAs (through diet choice, *de novo* biosynthesis, conversion, and/or selective mobilization from fat depots) may be a better way to optimize migratory performance than increasing the overall absolute FA levels during the day.

### 4.3 Fatty acids in relation to body fat

Generally, it is crucial to quickly build up fat stores at stopover sites for migrating birds. However, migrating blackbirds have relatively long stopovers and this species is a short‐distance migrant. Thus, it was not surprising that there was no difference in body fat score between migrants and residents. As mentioned above, the birds may have reached this condition through different dietary sources, or by feeding from the same sources but in different proportions. Regarding FAs and body fat score, a general pattern appeared for some of the FAs. The relative levels of the two essential PUFAs α‐linolenic acid and linoleic acid (along with arachidonic acid, DPA and pooled PUFA groups) were overall negatively related to body fat score, whereas both relative and absolute levels of MUFAs were positively related to body fat score. Thus, not only migrating birds have higher relative levels of PUFAs, but also lean birds overall. In a previous study on great tits, a similar pattern between body condition and FAs was revealed, with a decrease of certain PUFAs with body condition and an increase with the abundant MUFA, oleic acid. However, these associations were season‐dependent and stronger during the cold winter months (Andersson et al., 2015). The significance of these findings needs to be further investigated.

## 5 CONCLUSIONS

In summary, we show that migrant blackbirds have higher relative and absolute levels of both ω‐3 and ω‐6 PUFAs than resident conspecifics. Variation in the essential α‐linolenic acid suggests that there could be differences between migrants and residents in their dietary preferences for ω‐3 PUFAs, which contributes to the observed variation in plasma PUFA composition. To which extent this reflects dietary differences at the stopover site where birds were sampled versus differences at the breeding grounds remains unknown. The lack of a difference in the degree of unsaturation between migrants and residents could possibly be explained by a mechanism that regulates the potential exercise‐enhancing effect of PUFAs and the constraint of increased risk for lipid peroxidation which increases oxidative stress. Future studies should further investigate the underlying mechanisms and the costs and benefits of increased PUFA levels in migratory birds.

## CONFLICT OF INTEREST

The authors declare that there are no competing interests associated with this manuscript.

## AUTHOR CONTRIBUTIONS

**Johan Kjellberg Jensen:** Data curation (equal); Formal analysis (equal); Investigation (equal); Methodology (equal); Validation (equal); Writing‐original draft (equal). **Caroline Isaksson:** Conceptualization (equal); Data curation (equal); Formal analysis (equal); Funding acquisition (equal); Investigation (equal); Methodology (equal); Project administration (equal); Supervision (equal); Validation (equal); Writing‐review & editing (equal). **Cas Eikenaar:** Conceptualization (equal); Data curation (equal); Funding acquisition (equal); Investigation (equal); Methodology (equal); Project administration (equal); Validation (equal); Writing‐review & editing (equal). **Martin N. Andersson:** Conceptualization (equal); Data curation (equal); Formal analysis (equal); Investigation (equal); Methodology (equal); Supervision (equal); Validation (equal); Writing‐review & editing (equal).

## Supporting information

Supplementary Material

## Data Availability

The fatty acid data underlying the results of this study have be archived in Dryad (https://doi.org/10.5061/dryad.x69p8czg8).
