# Supplementary material for: Migrant blackbirds, Turdus merula, have higher plasma levels of polyunsaturated fatty acids compared to residents, but not enhanced fatty acid unsaturation index
Source: Ecol Evol. 2020 Aug 17;10(18):10196–206. doi: 10.1002/ece3.6681 (PMC7520213; doi:10.1002/ece3.6681)
Supplement: Supplementary file 1 — Supplementary Material [file ECE3-10-10196-s001.docx]

***Appendix 1.*** Summary table of the generalized linear models (GLMs) performed. **a)** Results for the relative levels (% of total fatty acid [FA] content) of all pooled FA classes and the unsaturation index. **b)** Results for the relative levels of all FAs tested individually. **c)** Results for the absolute concentration (ng/μl) of all pooled FA classes. Data are shown for the factors status (migrant or resident), body fat score (0-8) and time (time of sampling), and the status × time interaction. Significant results are highlighted in bold. See Methods for further details. SFAs = saturated fatty acids, MUFAs = monounsaturated fatty acids, PUFAs = polyunsaturated fatty acids

***Appendix 2.*** Summary table of additional generalized linear models (GLMs) performed, including the factors sex and age. ***a)*** Results for the relative levels (% of total fatty acid [FA] content) of all pooled FA classes and the unsaturation index. ***b)*** Results for the relative levels of all FAs tested individually. ***c)*** Results for the absolute concentration (ng/μl) of all pooled FA classes. Data are shown for the factors status (migrant or resident), body fat score (0-8), age, sex, and time (time of sampling), and the status × time interaction. Significant results are highlighted in bold. See Methods for further details. SFAs = saturated fatty acids, MUFAs = monounsaturated fatty acids, PUFAs = polyunsaturated fatty acids.

**Appendix 1**

| (a) | **Coefficients** | **Estimate** | **df** | | **F-value** | **p-value** |
| --- | --- | --- | --- | --- | --- | --- |
| Unsaturation index | \| Status \| \| --- \| \| Body fat \| \| Time \| \| Status $\times$ Time \| | \| -0.033 \| \| --- \| \| -0.021 \| \| -5.438e-06 \| \| -4.231e-05 \| | | \| 1 \| \| --- \| \| 1 \| \| 1 \| \| 1 \| | \| 0.468 \| \| --- \| \| 0.817 \| \| 0.001 \| \| 0.030 \| | \| 0.497 \| \| --- \| \| 0.370 \| \| 0.974 \| \| 0.863 \| |
| **Total PUFAs** | \| **Status** \| \| --- \| \| **Body fat** \| \| Time \| \| Status $\times$ Time \| | \| -0.086 \| \| --- \| \| -0.056 \| \| -0.0002 \| \| -0.0002 \| | | \| 1 \| \| --- \| \| 1 \| \| 1 \| \| 1 \| | \| 5.441 \| \| --- \| \| 10.216 \| \| 3.733 \| \| 0.033 \| | \| **0.023** \| \| --- \| \| **0.002** \| \| 0.058 \| \| 0.208 \| |
| **Total ω*-*3 PUFAs** | \| **Status** \| \| --- \| \| **Body fat** \| \| Time \| \| Status $\times$ Time \| | \| -0.011 \| \| --- \| \| -2.797e-05 \| \| -0.007 \| \| -2.890e-05 \| | | \| 1 \| \| --- \| \| 1 \| \| 1 \| \| 1 \| | \| 7.391 \| \| --- \| \| 12.841 \| \| 3.826 \| \| 1.918 \| | \| **0.008** \| \| --- \| \| **0.0007** \| \| 0.055 \| \| 0.171 \| |
| **Total ω*-*6 PUFAs** | \| **Status** \| \| --- \| \| **Body fat** \| \| Time \| \| Status $\times$ Time \| | \| -0.024 \| \| --- \| \| -0.016 \| \| -5.618e-05 \| \| -6.360e-05 \| | | \| 1 \| \| --- \| \| 1 \| \| 1 \| \| 1 \| | \| 4.056 \| \| --- \| \| 8.413 \| \| 1.835 \| \| 1.104 \| | \| **0.048** \| \| --- \| \| **0.005** \| \| 0.180 \| \| 0.297 \| |
| Total MUFAs | \| Status \| \| --- \| \| **Body fat** \| \| Time \| \| Status $\times$ Time \| | \| 0.023 \| \| --- \| \| 0.051 \| \| 9.242e-06 \| \| 1.461e-04 \| | | \| 1 \| \| --- \| \| 1 \| \| 1 \| \| 1 \| | \| 0.549 \| \| --- \| \| 11.665 \| \| 0.007 \| \| 0.825 \| | \| 0.461 \| \| --- \| \| **0.001** \| \| 0.933 \| \| 0.367 \| |
| **Total SFAs** | \| **Status** \| \| --- \| \| Body fat \| \| **Time** \| \| Status $\times$ Time \| | \| 0.039 \| \| --- \| \| -0.008 \| \| 1.395e-04 \| \| 3.663e-05 \| | | \| 1 \| \| --- \| \| 1 \| \| 1 \| \| 1 \| | \| 4.049 \| \| --- \| \| 0.727 \| \| 4.235 \| \| 0.137 \| | \| **0.048** \| \| --- \| \| 0.397 \| \| **0.044** \| \| 0.712 \| |

| (b) | **Coefficients** | **Estimate** | **df** | **F-value** | **p-value** |
| --- | --- | --- | --- | --- | --- |
| Palmitic acid 16:0  (SFA) | \| Status \| \| --- \| \| Body fat \| \| **Time** \| \| Status $\times$ Time \| | \| 0.020 \| \| --- \| \| -0.005 \| \| 2.076e-04 \| \| 5.551e-05 \| | \| 1 \| \| --- \| \| 1 \| \| 1 \| \| 1 \| | \| 0.957 \| \| --- \| \| 0.259 \| \| 8.419 \| \| 0.283 \| | \| 0.332 \| \| --- \| \| 0.612 \| \| **0.005** \| \| 0.597 \| |
| Palmitoleic acid  16:1n-7  (MUFA) | \| Status \| \| --- \| \| Body fat \| \| Time \| \| **Status** $\boldsymbol{\times}$ **Time** \| | \| 0.005 \| \| --- \| \| 0.035 \| \| 8.174e-05 \| \| 5.762e-04 \| | \| 1 \| \| --- \| \| 1 \| \| 1 \| \| 1 \| | \| 0.012 \| \| --- \| \| 2.778 \| \| 0.279 \| \| 6.502 \| | \| 0.913 \| \| --- \| \| 0.101 \| \| 0.599 \| \| **0.013** \| |
| **Stearic acid**  **18:0  (SFA)** | \| **Status** \| \| --- \| \| Body fat \| \| Time \| \| Status $\times$ Time \| | \| 0.044 \| \| --- \| \| -0.008 \| \| -9.786e-05 \| \| -1.384e-05 \| | \| 1 \| \| --- \| \| 1 \| \| 1 \| \| 1 \| | \| 5.364 \| \| --- \| \| 0.707 \| \| 2.123 \| \| 0.020 \| | \| **0.024** \| \| --- \| \| 0.404 \| \| 0.150 \| \| 0.888 \| |
| *cis*-Vaccenic acid  18:1n-7  (MUFA) | \| Status \| \| --- \| \| Body fat \| \| Time \| \| Status $\times$ Time \| | \| -0.011 \| \| --- \| \| -0.019 \| \| -1.187e-04 \| \| 9.356e-05 \| | \| 1 \| \| --- \| \| 1 \| \| 1 \| \| 1 \| | \| 0.181 \| \| --- \| \| 2.303 \| \| 1.645 \| \| 0.480 \| | \| 0.672 \| \| --- \| \| 0.134 \| \| 0.204 \| \| 0.491 \| |
| Oleic acid  18:1n-9  (MUFA) | \| Status \| \| --- \| \| **Body fat** \| \| Time \| \| Status $\times$ Time \| | \| 0.022 \| \| --- \| \| 0.056 \| \| 9.707e-06 \| \| 8.271e-05 \| | \| 1 \| \| --- \| \| 1 \| \| 1 \| \| 1 \| | \| 0.454 \| \| --- \| \| 12.331 \| \| 0.007 \| \| 0.235 \| | \| 0.503 \| \| --- \| \| **0.0008** \| \| 0.934 \| \| 0.630 \| |
| **α-Linolenic acid 18:3n-3 (ω-3 PUFA)** | \| **Status** \| \| --- \| \| **Body fat** \| \| Time \| \| Status $\times$ Time \| | \| -0.290 \| \| --- \| \| -0.105 \| \| 0.0001 \| \| 0.0006 \| | \| 1 \| \| --- \| \| 1 \| \| 1 \| \| 1 \| | \| 21.138 \| \| --- \| \| 12.085 \| \| 0.439 \| \| 3.465 \| | \| **< 0.0001** \| \| --- \| \| **0.0009** \| \| 0.510 \| \| 0.067 \| |
| **Eicosapentaenoic acid (EPA)**  **20:5n-3 (ω-3 PUFA)** | \| **Status** \| \| --- \| \| Body fat \| \| Time \| \| **Status** $\boldsymbol{\times}$ **Time** \| | \| -0.086 \| \| --- \| \| -0.027 \| \| -0.0002 \| \| -0.00038 \| | \| 1 \| \| --- \| \| 1 \| \| 1 \| \| 1 \| | \| 6.821 \| \| --- \| \| 2.952 \| \| 3.305 \| \| 4.950 \| | \| **0.011** \| \| --- \| \| 0.091 \| \| 0.074 \| \| **0.030** \| |
| **Docosapentaenoic acid (DPA)   22:5n-3 (ω-3 PUFA)** | \| **Status** \| \| --- \| \| **Body fat** \| \| Time \| \| **Status** $\boldsymbol{\times}$ **Time** \| | \| -0.101 \| \| --- \| \| -0.056 \| \| -0.0001 \| \| -0.0005 \| | \| 1 \| \| --- \| \| 1 \| \| 1 \| \| 1 \| | \| 5.297 \| \| --- \| \| 7.074 \| \| 0.449 \| \| 5.139 \| | \| **0.025** \| \| --- \| \| **0.010** \| \| 0.505 \| \| **0.027** \| |
| Docosahexaenoic acid (DHA)  22:6n-3  (ω-3 PUFA) | \| Status \| \| --- \| \| Body fat \| \| **Time** \| \| Status $\times$ Time \| | \| -0.012 \| \| --- \| \| -0.022 \| \| -0.0003 \| \| -0.0002 \| | \| 1 \| \| --- \| \| 1 \| \| 1 \| \| 1 \| | \| 0.133 \| \| --- \| \| 1.920 \| \| 7.986 \| \| 1.529 \| | \| 0.716 \| \| --- \| \| 0.171 \| \| **0.006** \| \| 0.221 \| |
| Linoleic acid 18:2n-6 (ω-6 PUFA) | \| Status \| \| --- \| \| **Body fat** \| \| Time \| \| Status $\times$ Time \| | \| -0.090 \| \| --- \| \| -0.077 \| \| -0.0003 \| \| 0.0003 \| | \| 1 \| \| --- \| \| 1 \| \| 1 \| \| 1 \| | \| 1.370 \| \| --- \| \| 4.377 \| \| 1.233 \| \| 0.560 \| | \| 0.246 \| \| --- \| \| **0.040** \| \| 0.271 \| \| 0.457 \| |
| Arachidonic acid  20:4n-6 (ω-6 PUFA) | \| Status \| \| --- \| \| **Body fat** \| \| **Time** \| \| **Status** $\boldsymbol{\times}$ **Time** \| | \| -0.055 \| \| --- \| \| -0.041 \| \| -2.320e-04 \| \| -4.115e-04 \| | \| 1 \| \| --- \| \| 1 \| \| 1 \| \| 1 \| | \| 3.928 \| \| --- \| \| 9.496 \| \| 5.656 \| \| 8.354 \| | \| 0.052 \| \| --- \| \| **0.003** \| \| **0.020** \| \| **0.005** \| |

| (c) | **Coefficients** | **Estimate** | **df** | **F-value** | **p-value** |
| --- | --- | --- | --- | --- | --- |
| Total FAs | \| Status \| \| --- \| \| Body fat \| \| Time \| \| Status $\times$ Time \| | \| -359.686 \| \| --- \| \| 158.842 \| \| -0.862 \| \| 1.566 \| | \| 1 \| \| --- \| \| 1 \| \| 1 \| \| 1 \| | \| 3.484 \| \| --- \| \| 2.973 \| \| 1.618 \| \| 2.512 \| | \| 0.067 \| \| --- \| \| 0.090 \| \| 0.208 \| \| 0.118 \| |
| **Total PUFAs** | \| **Status** \| \| --- \| \| Body fat \| \| **Time** \| \| Status $\times$ Time \| | \| -129.827 \| \| --- \| \| 8.758 \| \| -0.401 \| \| 0.326 \| | \| 1 \| \| --- \| \| 1 \| \| 1 \| \| 1 \| | \| 5.892 \| \| --- \| \| 0.117 \| \| 4.539 \| \| 1.409 \| | \| **0.018** \| \| --- \| \| 0.733 \| \| **0.037** \| \| 0.240 \| |
| **Total ω*-*3 PUFAs** | \| **Status** \| \| --- \| \| Body fat \| \| **Time** \| \| Status $\times$ Time \| | \| -40.781 \| \| --- \| \| 3.285 \| \| -0.106 \| \| 0.076 \| | \| 1 \| \| --- \| \| 1 \| \| 1 \| \| 1 \| | \| 8.175 \| \| --- \| \| 0.232 \| \| 4.485 \| \| 1.081 \| | \| **0.006** \| \| --- \| \| 0.632 \| \| **0.038** \| \| 0.302 \| |
| **Total ω*-*6 PUFAs** | \| **Status** \| \| --- \| \| Body fat \| \| **Time** \| \| Status $\times$ Time \| | \| -95.843 \| \| --- \| \| 3.594 \| \| -0.287 \| \| 0.249 \| | \| 1 \| \| --- \| \| 1 \| \| 1 \| \| 1 \| | \| 5.610 \| \| --- \| \| 0.035 \| \| 4.077 \| \| 1.439 \| | \| **0.021** \| \| --- \| \| 0.853 \| \| **0.048** \| \| 0.235 \| |
| Total MUFAs | \| Status \| \| --- \| \| **Body fat** \| \| **Time** \| \| Status $\times$ Time \| | \| -125.218 \| \| --- \| \| 92.387 \| \| -0.279 \| \| 0.647 \| | \| 1 \| \| --- \| \| 1 \| \| 1 \| \| 1 \| | \| 2.615 \| \| --- \| \| 6.228 \| \| 1.052 \| \| 2.656 \| | \| 0.111 \| \| --- \| \| **0.015** \| \| **0.309** \| \| 0.108 \| |
| Total SFAs | \| Status \| \| --- \| \| Body fat \| \| Time \| \| Status $\times$ Time \| | \| -99.293 \| \| --- \| \| 58.338 \| \| -0.188 \| \| 0.592 \| | \| 1 \| \| --- \| \| 1 \| \| 1 \| \| 1 \| | \| 1.950 \| \| --- \| \| 2.945 \| \| 0.568 \| \| 2.632 \| | \| 0.168 \| \| --- \| \| 0.091 \| \| 0.454 \| \| 0.110 \| |

**Appendix 2**

| (a) | **Coefficients** | **Estimate** | **df** | **F-value** | **p-value** |
| --- | --- | --- | --- | --- | --- |
| Unsaturation index | \| Status \| \| --- \| \| Body fat \| \| Time \| \| Age \| \| Sex \| \| Status $\times$ Time \| | \| -0.030 \| \| --- \| \| -0.016 \| \| -1.641e-06 \| \| 0.004 \| \| -0.080 \| \| -1.003e-04 \| | \| 1 \| \| --- \| \| 1 \| \| 1 \| \| 1 \| \| 1 \| \| 1 \| | \| 0.364 \| \| --- \| \| 0.462 \| \| 0.0001 \| \| 0.006 \| \| 2.903 \| \| 0.166 \| | \| 0.548 \| \| --- \| \| 0.499 \| \| 0.992 \| \| 0.938 \| \| 0.095 \| \| 0.685 \| |
| Total PUFAs | \| Status \| \| --- \| \| **Body fat** \| \| **Time** \| \| Age \| \| Sex \| \| Status $\times$ Time \| | \| -0.073 \| \| --- \| \| -0.055 \| \| -0.0003 \| \| -0.045 \| \| -0.0022 \| \| -0.0002 \| | \| 1 \| \| --- \| \| 1 \| \| 1 \| \| 1 \| \| 1 \| \| 1 \| | \| 3.590 \| \| --- \| \| 9.501 \| \| 4.213 \| \| 1.346 \| \| 0.004 \| \| 1.443 \| | \| 0.063 \| \| --- \| \| **0.003** \| \| **0.044** \| \| 0.251 \| \| 0.953 \| \| 0.234 \| |
| **Total ω*-*3 PUFAs** | \| **Status** \| \| --- \| \| **Body fat** \| \| Time \| \| Age \| \| Sex \| \| Status $\times$ Time \| | \| -0.010 \| \| --- \| \| -0.007 \| \| -2.917e-05 \| \| -0.003 \| \| 2.576e-04 \| \| -2.801e-05 \| | \| 1 \| \| --- \| \| 1 \| \| 1 \| \| 1 \| \| 1 \| \| 1 \| | \| 5.734 \| \| --- \| \| 12.106 \| \| 3.990 \| \| 0.416 \| \| 0.004 \| \| 1.718 \| | \| **0.020** \| \| --- \| \| **0.0009** \| \| 0.050 \| \| 0.521 \| \| 0.950 \| \| 0.195 \| |
| Total ω*-*6 PUFAs | \| Status \| \| --- \| \| **Body fat** \| \| Time \| \| Age \| \| Sex \| \| Status $\times$ Time \| | \| -0.020 \| \| --- \| \| -0.016 \| \| -6.160e-05 \| \| -0.013 \| \| -0.003 \| \| -6.229e-05 \| | \| 1 \| \| --- \| \| 1 \| \| 1 \| \| 1 \| \| 1 \| \| 1 \| | \| 2.611 \| \| --- \| \| 7.672 \| \| 2.141 \| \| 1.064 \| \| 0.054 \| \| 1.022 \| | \| 0.111 \| \| --- \| \| **0.007** \| \| 0.149 \| \| 0.306 \| \| 0.817 \| \| 0.316 \| |
| Total MUFAs | \| Status \| \| --- \| \| **Body fat** \| \| Time \| \| Age \| \| Sex \| \| Status $\times$ Time \| | \| 0.018 \| \| --- \| \| 0.050 \| \| 1.593e-05 \| \| 0.016 \| \| 0.008 \| \| 1.474e-04 \| | \| 1 \| \| --- \| \| 1 \| \| 1 \| \| 1 \| \| 1 \| \| 1 \| | \| 0.306 \| \| --- \| \| 10.757 \| \| 0.020 \| \| 0.235 \| \| 0.058 \| \| 0.800 \| | \| 0.582 \| \| --- \| \| **0.002** \| \| 0.888 \| \| 0.623 \| \| 0.810 \| \| 0.375 \| |
| **Total SFAs** | \| Status \| \| --- \| \| Body fat \| \| **Time** \| \| Age \| \| Sex \| \| Status $\times$ Time \| | \| 0.036 \| \| --- \| \| -0.008 \| \| 1.439e-04 \| \| 0.010 \| \| -0.005 \| \| 3.070e-05 \| | \| 1 \| \| --- \| \| 1 \| \| 1 \| \| 1 \| \| 1 \| \| 1 \| | \| 3.152 \| \| --- \| \| 0.681 \| \| 4.315 \| \| 0.242 \| \| 0.060 \| \| 0.092 \| | \| 0.081 \| \| --- \| \| 0.412 \| \| **0.042** \| \| 0.625 \| \| 0.808 \| \| 0.763 \| |

| (b) | **Coefficients** | **Estimate** | **df** | **F-value** | **p-value** |
| --- | --- | --- | --- | --- | --- |
| Palmitic acid 16:0  (SFA) | \| Status \| \| --- \| \| Body fat \| \| **Time** \| \| Age \| \| Sex \| \| Status $\times$ Time \| | \| 0.018 \| \| --- \| \| -0.005 \| \| 2.099e-04 \| \| 0.006 \| \| 0.004 \| \| 5.663e-05 \| | \| 1 \| \| --- \| \| 1 \| \| 1 \| \| 1 \| \| 1 \| \| 1 \| | \| 0.706 \| \| --- \| \| 0.285 \| \| 8.219 \| \| 0.072 \| \| 0.032 \| \| 0.279 \| | \| 0.404 \| \| --- \| \| 0.595 \| \| **0.006** \| \| 0.790 \| \| 0.859 \| \| 0.599 \| |
| Palmitoleic acid  16:1n-7  (MUFA) | \| Status \| \| --- \| \| Body fat \| \| Time \| \| Age \| \| Sex \| \| **Status** $\boldsymbol{\times}$ **Time** \| | \| 0.008 \| \| --- \| \| 0.035 \| \| 7.558e-05 \| \| -0.014 \| \| 0.012 \| \| 5.881e-04 \| | \| 1 \| \| --- \| \| 1 \| \| 1 \| \| 1 \| \| 1 \| \| 1 \| | \| 0.030 \| \| --- \| \| 2.578 \| \| 0.228 \| \| 0.085 \| \| 0.072 \| \| 6.430 \| | \| 0.863 \| \| --- \| \| 0.114 \| \| 0.635 \| \| 0.771 \| \| 0.790 \| \| **0.014** \| |
| **Stearic acid**  **18:0  (SFA)** | \| **Status** \| \| --- \| \| Body fat \| \| Time \| \| Age \| \| Sex \| \| Status $\times$ Time \| | \| 0.041 \| \| --- \| \| -0.007 \| \| -9.170e-05 \| \| 0.013 \| \| -0.021 \| \| -3.221e-05 \| | \| 1 \| \| --- \| \| 1 \| \| 1 \| \| 1 \| \| 1 \| \| 1 \| | \| 4.331 \| \| --- \| \| 0.512 \| \| 1.822 \| \| 0.424 \| \| 1.239 \| \| 0.105 \| | \| **0.042** \| \| --- \| \| 0.477 \| \| 0.182 \| \| 0.517 \| \| 0.270 \| \| 0.747 \| |
| *cis*-Vaccenic acid  18:1n-7  (MUFA) | \| Status \| \| --- \| \| Body fat \| \| Time \| \| Age \| \| Sex \| \| Status $\times$ Time \| | \| -0.003 \| \| --- \| \| -0.021 \| \| -1.357e-04 \| \| -0.038 \| \| 0.039 \| \| 1.309e-04 \| | \| 1 \| \| --- \| \| 1 \| \| 1 \| \| 1 \| \| 1 \| \| 1 \| | \| 0.009 \| \| --- \| \| 2.750 \| \| 2.184 \| \| 1.900 \| \| 2.322 \| \| 0.948 \| | \| 0.925 \| \| --- \| \| 0.102 \| \| 0.145 \| \| 0.173 \| \| 0.133 \| \| 0.334 \| |
| Oleic acid  18:1n-9  (MUFA) | \| Status \| \| --- \| \| **Body fat** \| \| Time \| \| Age \| \| Sex \| \| Status $\times$ Time \| | \| 0.017 \| \| --- \| \| 0.055 \| \| 1.873e-05 \| \| 0.021 \| \| -0.001 \| \| 7.640e-05 \| | \| 1 \| \| --- \| \| 1 \| \| 1 \| \| 1 \| \| 1 \| \| 1 \| | \| 0.222 \| \| --- \| \| 11.607 \| \| 0.025 \| \| 0.354 \| \| 0.002 \| \| 0.191 \| | \| 0.639 \| \| --- \| \| **0.001** \| \| 0.876 \| \| 0.554 \| \| 0.968 \| \| 0.664 \| |
| **α-Linolenic acid 18:3n-3 (ω-3 PUFA)** | \| **Status** \| \| --- \| \| **Body fat** \| \| Time \| \| **Age** \| \| Sex \| \| Status $\times$ Time \| | \| -0.250 \| \| --- \| \| 8.531e-05 \| \| -0.102 \| \| -0.147 \| \| -4.311e-04 \| \| 6.395e-04 \| | \| 1 \| \| --- \| \| 1 \| \| 1 \| \| 1 \| \| 1 \| \| 1 \| | \| 15.059 \| \| --- \| \| 11.628 \| \| 0.152 \| \| 5.007 \| \| 0.0000 \|   3.994 | \| **0.0003** \| \| --- \| \| **0.001** \| \| 0.698 \| \| **0.029** \| \| 0.994 \| \| 0.050 \| |
| **Eicosapentaenoic acid (EPA)**  **20:5n-3 (ω-3 PUFA)** | \| **Status** \| \| --- \| \| Body fat \| \| Time \| \| Age \| \| Sex \| \| **Status** $\boldsymbol{\times}$ **Time** \| | \| -0.084 \| \| --- \| \| -0.026 \| \| -0.0002 \| \| -0.006 \| \| -0.007 \| \| -0.0004 \| | \| 1 \| \| --- \| \| 1 \| \| 1 \| \| 1 \| \| 1 \| \| 1 \| | \| 5.787 \| \| --- \| \| 2.686 \| \| 3.228 \| \| 0.033 \| \| 0.042 \| \| 4.774 \| | \| **0.019** \| \| --- \| \| 0.106 \| \| 0.077 \| \| 0.857 \| \| 0.839 \| \| **0.033** \| |
| **Docosapentaenoic acid (DPA)   22:5n-3 (ω-3 PUFA)** | \| Status \| \| --- \| \| **Body fat** \| \| Time \| \| Age \| \| Sex \| \| **Status** $\boldsymbol{\times}$ **Time** \| | \| -0.088 \| \| --- \| \| -0.0001 \| \| -0.057 \| \| -0.055 \| \| 0.040 \| \| -0.0005 \| | \| 1 \| \| --- \| \| 1 \| \| 1 \| \| 1 \| \| 1 \| \| 1 \| | \| 3.685 \| \| --- \| \| 7.286 \| \| 0.673 \| \| 1.382 \| \| 0.858 \| \| 4.234 \| | \| 0.060 \| \| --- \| \| **0.009** \| \| 0.415 \| \| 0.244 \| \| 0.358 \| \| **0.044** \| |
| Docosahexaenoic acid (DHA)  22:6n-3  (ω-3 PUFA) | \| Status \| \| --- \| \| Body fat \| \| **Time** \| \| Age \| \| Sex \| \| Status $\times$ Time \| | \| -0.014 \| \| --- \| \| -0.021 \| \| -0.0003 \| \| 0.010 \| \| -0.021 \| \| -0.0002 \| | \| 1 \| \| --- \| \| 1 \| \| 1 \| \| 1 \| \| 1 \| \| 1 \| | \| 0.157 \| \| --- \| \| 1.655 \| \| 7.443 \| \| 0.079 \| \| 0.392 \| \| 1.709 \| | \| 0.693 \| \| --- \| \| 0.203 \| \| **0.008** \| \| 0.779 \| \| 0.533 \| \| 0.196 \| |
| Linoleic acid 18:2n-6 (ω-6 PUFA) | \| Status \| \| --- \| \| **Body fat** \| \| Time \| \| Age \| \| Sex \| \| Status $\times$ Time \| | \| -0.070 \| \| --- \| \| -0.078 \| \| -0.0003 \| \| -0.077 \| \| 0.044 \| \| 0.0003 \| | \| 1 \| \| --- \| \| 1 \| \| 1 \| \| 1 \| \| 1 \| \| 1 \| | \| 0.768 \| \| --- \| \| 4.350 \| \| 1.480 \| \| 0.878 \| \| 0.324 \| \| 0.741 \| | \| 0.384 \| \| --- \| \| **0.041** \| \| 0.228 \| \| 0.353 \| \| 0.571 \| \| 0.393 \| |
| Arachidonic acid  20:4n-6 (ω-6 PUFA) | \| Status \| \| --- \| \| **Body fat** \| \| **Time** \| \| Age \| \| Sex \| \| **Status** $\boldsymbol{\times}$ **Time** \| | \| -0.047 \| \| --- \| \| -0.039 \| \| -0.0002 \| \| -0.028 \| \| -0.014 \| \| -0.0004 \| | \| 1 \| \| --- \| \| 1 \| \| 1 \| \| 1 \| \| 1 \| \| 1 \| | \| 2.567 \| \| --- \| \| 8.511 \| \| 6.038 \| \| 0.864 \| \| 0.258 \| \| 8.187 \| | \| 0.114 \| \| --- \| \| **0.005** \| \| **0.017** \| \| 0.356 \| \| 0.613 \| \| **0.006** \| |

| (c) | **Coefficients** | **Estimate** | **df** | **F-value** | **p-value** |
| --- | --- | --- | --- | --- | --- |
| Total FAs | \| Status \| \| --- \| \| Body fat \| \| Time \| \| Age \| \| Sex \| \| Status $\times$ Time \| | \| -356.084 \| \| --- \| \| 172.535 \| \| -0.845 \| \| 25.957 \| \| -221.514 \| \| 1.402 \| | \| 1 \| \| --- \| \| 1 \| \| 1 \| \| 1 \| \| 1 \| \|  \| | \| 3.107 \| \| --- \| \| 3.403 \| \| 1.514 \| \| 0.016 \| \| 1.329 \| \| 1.947 \| | \| 0.083 \| \| --- \| \| 0.070 \| \| 0.223 \| \| 0.900 \| \| 0.254 \| \| 0.168 \| |
| **Total PUFAs** | \| **Status** \| \| --- \| \| Body fat \| \| **Time** \| \| **Age** \| \| **Sex** \| \| Status $\times$ Time \| | \| -118.194 \| \| --- \| \| 12.919 \| \| -0.413 \| \| -32.686 \| \| -52.818 \| \| 0.296 \| | \| 1 \| \| --- \| \| 1 \| \| 1 \| \| 1 \| \| 1 \| \| 1 \| | \| 4.447 \| \| --- \| \| 0.248 \| \| 4.699 \| \| 0.327 \| \| 0.981 \| \| 1.129 \| | \| **0.039** \| \| --- \| \| 0.620 \| \| **0.034** \| \| **0.570** \| \| **0.326** \| \| 0.292 \| |
| **Total ω*-*3 PUFAs** | \| **Status** \| \| --- \| \| Body fat \| \| **Time** \| \| Age \| \| Sex \| \| Status $\times$ Time \| | \| -37.169 \| \| --- \| \| 4.460 \| \| -0.110 \| \| -10.486 \| \| -14.472 \| \| 0.068 \| | \| 1 \| \| --- \| \| 1 \| \| 1 \| \| 1 \| \| 1 \| \| 1 \| | \| 6.206 \| \| --- \| \| 0.417 \| \| 4.724 \| \| 0.475 \| \| 1.040 \| \| 0.850 \| | \| **0.015** \| \| --- \| \| 0.521 \| \| **0.034** \| \| 0.494 \| \| 0.312 \| \| 0.360 \| |
| **Total ω*-*6 PUFAs** | \| **Status** \| \| --- \| \| Body fat \| \| **Time** \| \| Age \| \| Sex \| \| Status $\times$ Time \| | \| -86.685 \| \| --- \| \| 6.743 \| \| -0.297 \| \| -26.098 \| \| -39.487 \| \| 0.227 \| | \| 1 \| \| --- \| \| 1 \| \| 1 \| \| 1 \| \| 1 \| \| 1 \| | \| 4.180 \| \| --- \| \| 0.118 \| \| 4.252 \| \| 0.364 \| \| 0.959 \| \| 1.163 \| | \| **0.045** \| \| --- \| \| 0.732 \| \| **0.043** \| \| 0.549 \| \| 0.331 \| \| 0.285 \| |
| Total MUFAs | \| Status \| \| --- \| \| **Body fat** \| \| Time \| \| Age \| \| Sex \| \| Status $\times$ Time \| | \| -132.325 \| \| --- \| \| 96.676 \| \| -0.260 \| \| 39.836 \| \| -80.704 \| \| 0.580 \| | \| 1 \| \| --- \| \| 1 \| \| 1 \| \| 1 \| \| 1 \| \| 1 \| | \| 2.654 \| \| --- \| \| 6.609 \| \| 0.889 \| \| 0.231 \| \| 1.091 \| \| 2.061 \| | \| 0.109 \| \| --- \| \| **0.013** \| \| 0.349 \| \| 0.632 \| \| 0.300 \| \| 0.156 \| |
| Total SFAs | \| Status \| \| --- \| \| Body fat \| \| Time \| \| Age \| \| Sex \| \| Status $\times$ Time \| | \| -101.225 \| \| --- \| \| 63.355 \| \| -0.177 \| \| 22.033 \| \| -85.645 \| \| 0.525 \| | \| 1 \| \| --- \| \| 1 \| \| 1 \| \| 1 \| \| 1 \| \| 1 \| | \| 1.849 \| \| --- \| \| 3.379 \| \| 0.488 \| \| 0.084 \| \| 1.463 \| \| 2.013 \| | \| 0.179 \| \| --- \| \| 0.071 \| \| 0.487 \| \| 0.772 \| \| 0.231 \| \| 0.161 \| |
